# Exstrophy Bladder with Low Anorectal Malformation- A Rare Association

**DOI:** 10.21699/jns.v6i3.494

**Published:** 2017-08-10

**Authors:** Aditya Pratap Singh, Vinay Mathur, Ramesh Tanger, Arun Kumar Gupta

**Affiliations:** Department of Surgery (Pediatric Surgery), SMS Medical College Jaipur, Rajsthan, India.

**Dear Sir**

A 4-day-old male newborn, weighing 2.5 Kg, delivered vaginally at 38 weeks presented with malformed external genitalia and a reddish swelling over the lower part of the abdomen and the absent anal opening with a small perineal opening. Pregnancy was uneventful. Maternal serologies for HbsAg, HCV, HIV, Toxoplasma, and Rubella were planned but due to financial reason not done. Physical examination revealed a lobulated red mass with lower abdominal wall defect. The umbilicus was low set, situated just above the upper extent of the swelling. The pubic symphysis was widely separated on X-ray and utrasound. Exposed, everted bladder template was clearly visible immediately below umbilical stump; a completely dorsally opened (epispadic) urethral plate run from bladder neck down to the open glans; left and right corpora cavernosa were clearly visible beneath and alongside urethral plate; the scrotum was normally developed, but caudally displaced; anus was absent at the normal site. Anal opening was present as anteriorly placed perineal fistula. Physical examination was otherwise unremarkable. The defect was covered with sterile saline gauzes. USG brain suggestive of a subependymal cyst with germinal matrix hemorrhage and prominent lenticulostrias arteries suggestive of TORCH. There were no urinay or GIT anomalies on USG abdomen. Anoplasty with primary closure of the bladder was done..

Exstrophy bladder in male patients usually comprised of high anorectal malformation. In our case there was low ARM. It is very rare to see associated anomalies in the classical exstrophy bladder. This rare congenital anomaly is thought to be a clinical spectrum ranging from isolated epispadias to classic bladder exstrophy (CBE), to its most severe form, cloacal exstrophy (CE) [1,2]. CE is also referred to as the ‘OEIS’ complex, an acronym for omphalocele, exstrophy of the bladder, imperforate anus and spinal defects [3]. Our case does not fit in the OEIS complex too owing to absent omphalocele and spinal defects. In our view, it is very rare case of exstrophy bladder with low ARM as an isolated associated anomaly. 

**Figure:**
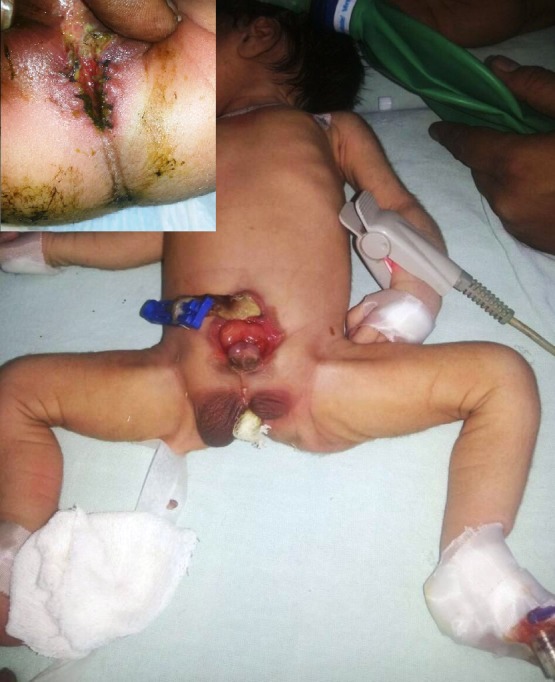
Figure 1: Showing exstrophy of bladder and epispadias. Inset shows low anorectal malformation.

## Footnotes

**Source of Support:** None

**Conflict of Interest:** None
